# Microwave-assisted synthesis of Ag/ZnO nanoparticles using *Averrhoa carambola* fruit extract as the reducing agent and their application in cotton fabrics with antibacterial and UV-protection properties

**DOI:** 10.1039/d2ra01636b

**Published:** 2022-05-18

**Authors:** Paweena Porrawatkul, Rungnapa Pimsen, Arnannit Kuyyogsuy, Nongyao Teppaya, Amnuay Noypha, Saksit Chanthai, Prawit Nuengmatcha

**Affiliations:** Creative Innovation in Science and Technology, Department of Chemistry, Faculty of Science and Technology, Nakhon Si Thammarat Rajabhat University 80280 Thailand pnuengmatcha@gmail.com prawit_nue@nstru.ac.th; Nanomaterials Chemistry Research Unit, Department of Chemistry, Faculty of Science and Technology, Nakhon Si Thammarat Rajabhat University 80280 Thailand; Materials Chemistry Research Center, Department of Chemistry and Center of Excellence for Innovation in Chemistry, Faculty of Science, Khon Kaen University Khon Kaen 40002 Thailand

## Abstract

This is the first time *Averrhoa carambola* fruit extract has been used as a reducing agent to synthesize Ag/ZnO composites for coating cotton to develop antibacterial activity and UV protection under domestic microwave irradiation. The effects of the molar concentration of silver nitrate solutions, applied power, reaction duration, and pH on the yield of nanoparticles were determined. The treated fabrics were subjected to the investigation of surface morphology and chemical structure using SEM and EDX techniques, respectively. The antibacterial activity of the ZnO NPs and the Ag/ZnO nanocomposite coated on cotton fabric was evaluated against *E. coli* and *S. aureus* using the agar well diffusion method. The results revealed good antibacterial activity in the cotton fabric treated with the Ag-doped ZnO composite. The stability of the Ag/ZnO nanocomposite coated fabrics was determined by a wash durability test, the results of which demonstrated that this fabric could retain good antibacterial activity even after 20 wash cycles. The UV-blocking capacity of the treated fabrics was evaluated based on the ultraviolet protection factor (UPF) value determined in the range of 280–400 nm. The UPF value determined for the Ag/ZnO-coated fabric was 69.67 ± 1.53, which indicated an excellent ability to block UV radiation. Collectively, these results demonstrated the Ag/ZnO nanocomposite prepared in the present study as a promising material for preparing textiles with good antibacterial activity and UV protection.

## Abstract 2

This is the first time *Averrhoa carambola* fruit extract has been used as a reducing agent to synthesize Ag/ZnO composites for coating cotton to develop antibacterial activity and UV protection under domestic microwave irradiation.

## Graphical abstract

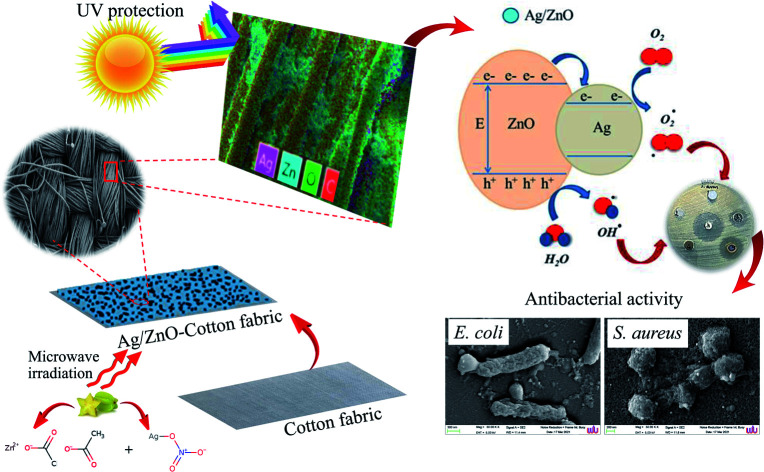

## 1. Introduction

Cotton fabric is popular among people owing to its excellent properties, such as elasticity, softness, water absorption, and air permeability. However, the fabric also favors the growth of germs, which reduces its wearability due to potential risks to human health.^1,2^ Several surface modification approaches have been reported to improve cotton fabric in terms of developing antibacterial properties, fire resistance, and UV protection in the prepared fabrics.^3–5^ One of these methods is the use of metal oxide nanoparticles, such as silver nanoparticles, zinc oxide nanoparticles, and titanium oxide nanoparticles.^6–8^ The characteristics mainly targeted to improve the cotton fabric are its antibacterial properties and UV protection. Nowadays, ZnO nanoparticles are being preferred to improve such properties, as these particles are non-toxic in nature and exhibit diverse chemical, physical, and biological properties. Moreover, ZnO nanoparticles exhibit a wide bandgap (3.37 eV), high binding energy (60 MeV), high chemical balance, and excellent photostability.^9^ The food and drug administration (FDA, USA) has even designated ZnO as a safe substance.^10,11^ ZnO is generally utilized as a bactericidal agent, inhibitor against both Gram-negative and Gram-positive bacteria, and a UV-protection substance.^12,13^ When semiconductor nanocrystals are exposed to light radiation with an energy value greater than their bandgap, electron–hole pairs are produced; electrons in the CB and holes in the VB. These produced holes are responsible for the oxidation of the organics. The adsorbed oxygen molecule absorbs the electron, forming a superoxide radical (O_2_˙^−^). In the presence of moisture, this O˙^−^ forms different reactive species, such as HO˙, HO_2_˙, and H_2_O_2_, that act as oxidants. However, when the holes are captured by surface hydroxyl groups or adsorbed water molecules, short-lived HO radicals are generated, which reduce the antibacterial activity and UV protection.^14^

ZnO nanoparticles doped with metals, such as Ag, Li, Fe, Co, Ni, Mn, and Cr, have been used widely for improving the antibacterial activity and UV protection of textiles.^15–17^ The nanocrystalline ZnO doped with Ag exhibited improved performance and activity and has, therefore, become a research hotspot in this field. Ag-doping of nanoparticles alters the optical and electrical properties of the material, which has an impact on the UV protection property of the material.^18^ ZnO nanoparticles are generally synthesized using various chemical and physical processes, such as microwave-assisted method, hydrothermal method, co-precipitation, electrochemical technique, ultrasonic approach, and sol–gel procedures.^19–24^ However, most of these processes involve the use of toxic chemicals and solvents that prove to be detrimental to both human health and the environment. Microwave-assisted synthesis of ZnO nanoparticles is a promising technique for fabricating nanostructures within a controlled environment reliably, less energy consumption and cost-effectively.^25–27^ Moreover, this technique is highly adjustable when nanostructures of particular types are to be synthesized for specific commercial purposes.^28^

Recently, the phytochemical method of synthesizing metal oxide nanoparticles has been gaining huge interest owing to the eco-friendliness, low cost, non-toxicity to humans, convenience of synthesis, and bio-inspiration of this method. Phytocompounds derived from plants, such as flavonoids, alkaloids, carotenoids, terpenoids, tannins, and chlorophyll, are employed as reducing and capping agents in the synthesis of ZnO NPs.^29^ Extracts from different plants have been applied successfully in the synthesis of ZnO nanoparticles, including *Capparis decidua*,^17^*Crataegus monogyna* fruit,^30^*Carya illinoinensis* leaf,^31^*Azadirachta indica* leaf,^32^ and *Trigonella oenum-graecum* leaf.^33^ However, to date, no study has reported the use of *Averrhoa carambola* fruit extract for the synthesis of Ag-doped ZnO composite using the microwave-assisted approach. *Averrhoa carambola* fruit or star fruit contains flavonoids, including anthocyanins and ellagitannins, in huge amounts, because of which its extract was expected to serve as an effective reducing agent. The star fruit extracts have previously been reported to have great potential as an antibacterial agent.^34^

In this context, the present study reports the domestic microwave-assisted bio-synthesis of Ag-doped ZnO nanoparticles (Ag/ZnO NPs) using the *Averrhoa carambola* extract as a reducing agent. The biosynthesis process was also optimized for applied power, reaction duration, the concentration of Ag used, and reaction pH. The synthesized Ag/ZnO nanocomposites were then subjected to powder X-ray diffraction, scanning electron microscopy, energy dispersive X-ray, transmission electron microscopy, and Fourier-transform infrared spectroscopy to determine their physicochemical properties. Cotton fabrics were modified using these synthesized Ag/ZnO NPs as a coating and then subjected to antibacterial tests against *Escherichia coli* (*E. coli*) and *Staphylococcus aureus* (*S. aureus*) using the agar well diffusion method. The UV protection properties of these cotton fabrics were also investigated.

## 2. Experimental

### 2.1 Materials and reagents

All reagents used in the present study were of analytical grade and were purchased from Merck. The nutrient agar for bacterial culture, the Mueller–Hinton broth, and the antimicrobial activity agar was purchased from Hi-Media (Mumbai, India). Cotton fabric (100%, 220 g m^−2^) was purchased from Chok-dee Textiles Company (Nakhon Si Thammarat province, Thailand).

### 2.2 Selection and preparation of *Averrhoa carambola* fruit extract

The samples of the star fruit from *Averrhoa carambola* were collected from Thailand's Ronpiboon district in the province of Nakhon Si Thammarat. The collected fruits were washed and dried in the shade, chopped into little pieces. In order to obtain the extracts from the fruits of *Averrhoa carambola*, 200 g of the cut pieces of fruits were homogenized in 200 mL of 95 percent ethanol for 3 h at room temperature. The homogenate was filtered through a Whatman No. 1 filter paper, and the solvent was removed from the filtrate using an evaporator. This crude extract was used as the reducing agent in subsequent experiments.

### 2.3 Synthesis of zinc oxide NPs and Ag-doped ZnO NPs

**Fig. 1:**
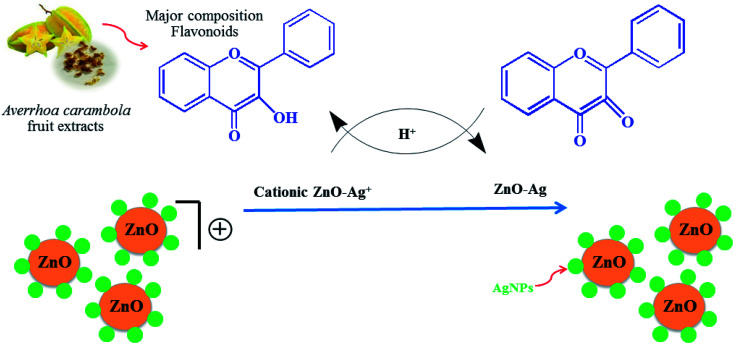
Schematic of the green synthesis of Ag/ZnO NPs using the microwave-assisted approach.

ZnO nanopowder was synthesized by dissolving 1.2 g of zinc acetate dihydrate (Zn(CH_3_CO_2_)_2_) and 0.5 g of the crude star fruit extract in 50 mL of DI water followed by constant stirring. Next, the pH (9, 10, 11, and 12) variable in the synthesis of ZnO nanoparticles was optimized first, and the pH of the blending solution was altered using 2 M NaOH. The next optimization experiment involved adding different concentrations (0.05, 0.07, 0.10, and 0.15 mol L^−1^) of silver nitrate hexahydrate (Ag(NO_3_)_2_·6H_2_O) to a solution of zinc acetate dehydrate under constant magnetic stirring. The power variable was optimized by treating the reaction mixture with a pressure-controlled microwave synthesizer set to different power levels of 300, 450, 600, and 700 W. The reaction duration was optimized under microwave irradiation for 3 min, 5 min, 8 min, and 10 min. In the experiments, the heated solution containing a white precipitate was allowed to cool naturally at room temperature. Subsequently, the white precipitate was collected through centrifugation at 4000 rpm for 10 min, washed several times with DI water and 100% ethyl alcohol to remove any remnants of the extract, and then dried in the air at 60 °C for 24 h. UV-Vis spectrophotometry was employed to investigate the optimal conditions for the synthesis process. The suggested mechanism for the preparation of Ag/ZnO NPs nanoparticles in presence of *Averrhoa carambola* fruit extract is presence of Fig. 1.

### 2.4 Coating of ZnO NPs and Ag/ZnO NPs on cotton fabrics

The synthesized Ag/ZnO NPs (0.5 g) was dissolved in 20 mL of chitosan solution (20 mg chitosan in 20 mL of 1 percent acetic acid) followed by stirring for 1 h at room temperature using a magnetic stirrer. The dispersion of Ag/ZnO NPs particles in the solution was promoted through 30 min of sonication. Approximately 4 g of bio-scoured cotton fabric was immersed in the Ag/ZnO NPs in chitosan solution for 30 min prior to being padded in the padding mangle and squeezing between two squeezing rollers for an 80 percent wet pick-up. The padded fabric was dried at 70 °C for 10 min and then cured at 150 °C for 5 min. The cured fabric was washed thoroughly in a 1% sodium hydroxide solution, neutralized with 1% acetic acid, washed once again, and then air-dried. Scanning electron microscopy (SEM) (Quanta 400 (SEM-Quanta)) was employed to analyze the surface morphology of uncoated and coated cotton fibers. Energy-dispersive X-ray spectroscopy (EDX) (Quanta 400 (SEM-Quanta)) was employed to investigate the elemental composition of the untreated cotton fiber and the cotton fabric coated with ZnO NPs and Ag/ZnO NPs.

### 2.5 Characterization of the biosynthesized ZnO and Ag/ZnO nanoparticles

The synthesis process of Ag/ZnO NPs was monitored visually. In addition, UV-Vis spectrophotometry was employed to measure the absorption spectra of the synthesized ZnO NPs. The surface plasmon resonance peak of the synthesized ZnO NPs and Ag/ZnO nanocomposite were measured through UV-visible spectrophotometry conducted in the wavelength range of 300–700 nm to determine the maximum absorbance values. The morphology and composition of the synthesized NPs and composite were analyzed using scanning electron microscopy (SEM) and X-ray Diffractometry (XRD) (Shimata; XD-D1). Furthermore, energy-dispersive X-ray spectroscopy (EDX) was employed to analyze the elemental distribution on the surface of samples. Particle size distributions in ZnO NPs and Ag/ZnO nanocomposite samples were determined using laser particle size analyzer (LPSA) (Beckman Coulter, LS 320). Transmission electron microscopy (TEM) (TEM, JEOL; JEM-2010) was employed to obtain the images of the Ag/ZnO nanocomposite. The presence of Ag nanocomposite on the ZnO nanoparticles was confirmed through Fourier-transform infrared spectroscopy (FT-IR) (Bruker, Germany, VERTEX).

### 2.6 Antibacterial activity analysis

The antibacterial activity of the *Averrhoa carambola* fruit extract, metal oxide NPs, and the modified cotton fabric against *Staphylococcus aureus* (*S. aureus*) and *Escherichia coli* (*E. coli*) was evaluated using the agar well diffusion method. A sterile cotton swab was used for disseminating the bacterial culture evenly across the nutrient agar plate. Subsequently, wells were prepared in the agar plates, and standard antibiotic discs (chloramphenicol) were added to these wells. The diameter of the inhibition zone surrounding the sample was measured and compared to that around the commercial standard antibiotic chloramphenicol and ethanoic extract after incubation at 37 °C for 24 h. The durability of the antibacterial activity after washing was determined by treating the modified fabrics with 1 g L^−1^ of laundry detergent at 35 °C for 20 min and then measuring the activity at the 5^th^, 10^th^, 15^th^, and 20^th^ washing cycles.

### 2.7 UV protection property analysis

The responses of the treated and untreated cotton fabrics to UV light were evaluated using UV-Vis spectroscopy (Thermo scientific, Evolution 201). The UV absorption and transmission were measured to determine the effectiveness of the UV shielding. The ultraviolet protection factor (UPF) and percentage UV transmission were calculated using the transmission data and relevant formulas. The UPF was calculated by dividing the average effective irradiance for skin by the average UV irradiation for the skin shielded by the fabric being evaluated; the mean% transmission in the UV range (280–400 nm) was used for this calculation (AATCC Test Method 183-2004).^35^
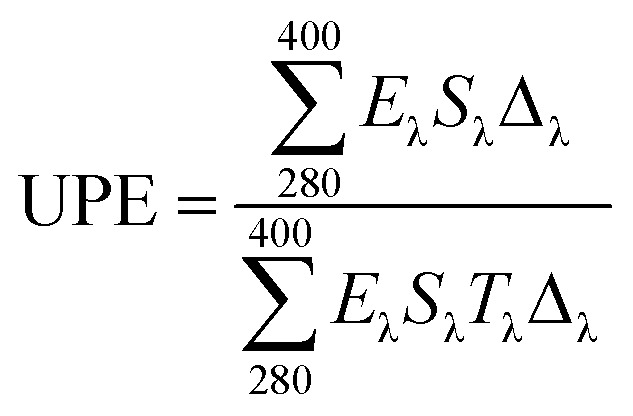


In the above equation, *E*_λ_ is the relative erythemal spectral effectiveness, *S*_λ_ is the solar spectral irradiance in W m^−2^ nm^−1^, and *T*_λ_ is the UV spectrometric spectral transmission specimen. *E*_λ_ and *S*_λ_ were obtained from a database maintained by the National Oceanic and Atmospheric Administration.

## 3. Results and discussion

### 3.1 Synthesis of Ag/ZnO nanoparticles

**Fig. 2:**
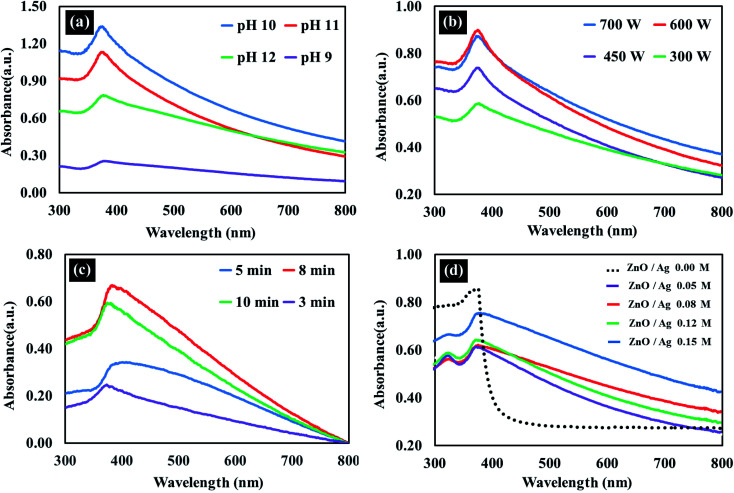
UV spectra illustrating the effect of (a) pH (b) applied power (c) reaction duration and (d) Ag ion concentration on the synthesis of Ag/ZnO nanoparticles.

In the present study, Ag-doped ZnO nanoparticles were synthesized using *Averrhoa carambola* fruit extract, which contains various phytochemicals, such as aliphatic amines, phenolic compounds, flavonoids, aromatic compounds, and alkyne groups,^36^ as its main constituents. After the addition of the *Averrhoa carambola* fruit extract to the zinc nitrate solution under alkaline conditions, the color of the reaction mixture changed from light yellow to white, which changed further to brown when Ag was added. This color change, which occurs due to surface plasmon resonance occurring within the synthesized metal nanoparticles, may be utilized for indicating the presence of ZnO nanoparticles. The electron donor groups present in the fruit extract of *Averrhoa carambola* could have been involved in this color conversion of the synthesized Ag/ZnO NPs. Moreover, the extract also served as a stabilizer for the formation of Ag/ZnO nanoparticles. A UV-Visible spectrophotometer was operated in the wavelength range of 250 to 800 nm to characterize the synthesized Ag/ZnO NPs. Fig. 2 provides the UV-vis absorption spectrum of all samples. The synthesized pure ZnO NPs have an absorption edge around 375 nm. It can be seen that Ag loaded ZnO samples exhibit the intense absorption in the visible light region (Fig. 2(a)). The enhanced visible light (>400 nm) absorption of Ag–ZnO nanoparticles is caused by the surface plasmon resonance (SPR) of metallic AgNPs, in particular.^37^

#### 3.1.1 Effect of pH on the synthesis of Ag/ZnO nanoparticles

In general, alkaline conditions favor the synthesis of metal oxides. In the present study, the synthesis process was observed at pH 9, 10, 11, and 12, and the resulting UV-Vis spectra of the corresponding synthesized Ag/ZnO NPs are depicted in Fig. 2(a). The spectrum obtained at pH 10 exhibited the highest absorbance. Therefore, pH 10 was selected as the optimal pH condition for the synthesis of Ag/ZnO nanoparticles.

#### 3.1.2 Effect of applied power on the synthesis of Ag/ZnO nanoparticles

Fig. 2(b) depicts the UV-Vis spectra of the Ag/ZnO NPs synthesized at 300 W, 450 W, 600 W, and 700 W. As evident, the absorbance of the Ag/ZnO nanocomposite increased with an increase in the applied power between 300 W to 400 W, beyond which the concentration of the synthesized product remained nearly the same until 700 W. At 700 W, the absorbance was again slightly reduced. Therefore, the power of 600 W was selected as the optimal power condition for the synthesis of Ag/ZnO NPs.

#### 3.1.3 Effect of reaction duration on the synthesis of Ag/ZnO nanoparticles

The reaction duration is another important factor affecting the product yield of a reaction. The UV spectra of the Ag/ZnO nanocomposite after the reaction duration of 3 min, 5 min, 8 min, and 10 min are depicted in Fig. 2(c). The peak representing the highest absorbance was obtained at the reaction duration of 8 min. At 10 min, the absorbance again decreased, which could be due to product decay. Therefore, the reaction duration of 8 min was selected as the optimum duration for the synthesis process of Ag/ZnO NPs. Synthesis from star fruit extracts in a microwave-assisted appropriate short time than previously reported.^38,39^

#### 3.1.4 Effect of Ag ion concentration on the synthesis of Ag/ZnO nanoparticles

Fig. 2(d) depicts the effect of Ag ion concentration on the synthesis of Ag/ZnO NPs. The spectrum of pure ZnO NPs exhibited a maximum absorbance of around 378 nm and transparence in the visible light region. An increase in the Ag ion concentration resulted in a steady increase in the absorbance around 375 nm and signal in visible light region, with the absorbance increasing as the duration of incubation increased in the Ag ion concentration range of 0.05–0.15 M. The UV-Visible spectra revealed the maximum absorbance at an Ag ion concentration of 0.15 M, which was then selected as the optimal Ag ion concentration for the synthesis of Ag/ZnO nanoparticles.

### 3.2 FT-IR analysis

**Fig. 3:**
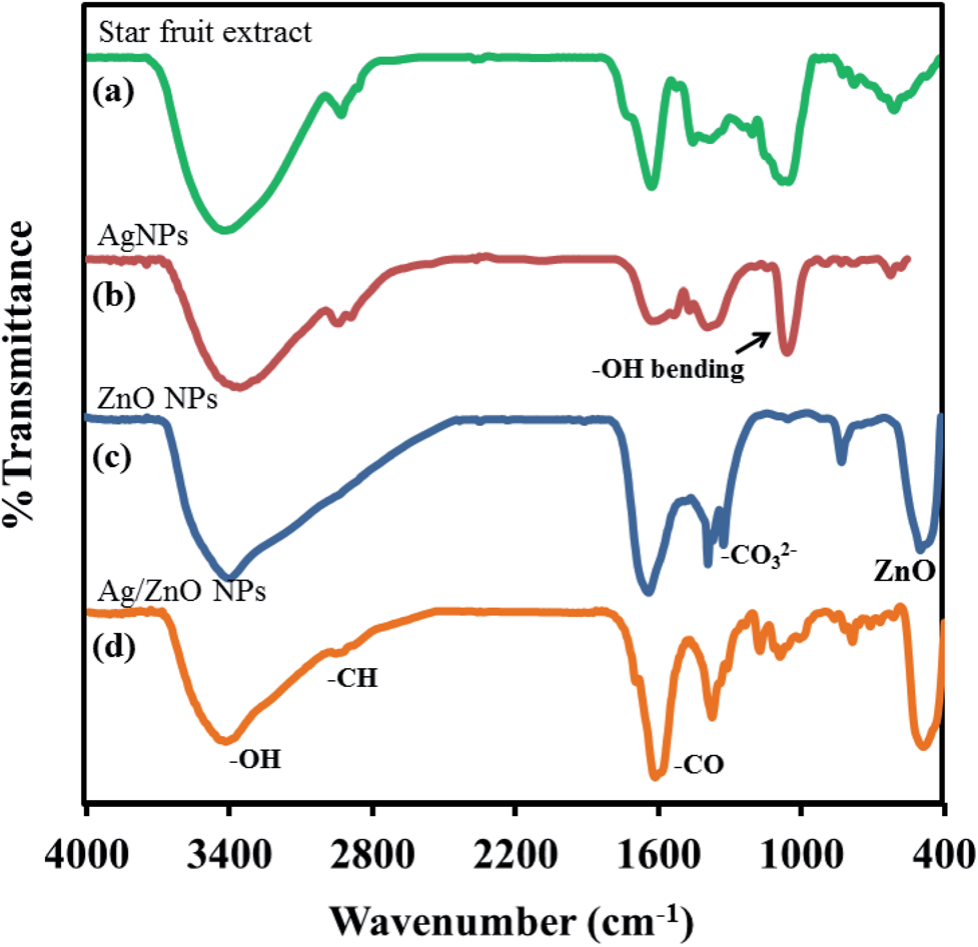
FTIR spectra of (a) the star fruit extract (b) AgNPs (c) ZnO NPs (d) Ag/ZnO NPs.

FT-IR spectral analysis was performed to confirm the successful synthesis of nanocrystalline ZnO wurtzite structure and also the influence of the addition of silver to ZnO. Fig. 3 presents a comparative analysis of the FTIR spectra of star fruit extract, AgNPs, ZnO NPs, and Ag/ZnO NPs. The FTIR spectra of star fruit extract, depicted in Fig. 3(a), presented the C–H stretching vibration of the –CH_3_ group at approximately 2900 cm^−1^. The broad spectral peak at approximately 3400 cm^−1^ represented the O–H stretching of alcohols.^40^ The sharp stretching vibration at 1653 cm^−1^ corresponded to the C=O functional groups. The FTIR spectrum of AgNPs exhibited decreased O–H and C–O stretching intensity (Fig. 3(b)), the C–O stretching peak at 1250 cm^−1^, and a broad peak for O–H stretching at 3300 cm^−1^, indicating the oxidation of the C=O group to the –COOH group.^41^ According to these FTIR data, the O–H and C=O groups in the star fruit extract were responsible for the production of silver nanoparticles by serving as the reducing and stabilizing agents, respectively.^42^ The broad peak at 3400 cm^−1^ detected in the ZnO NPs FTIR spectrum depicted in Fig. 3 corresponded to the stretching vibration of O–H in the water molecules chemisorbed onto the ZnO surface. The band at 2900 cm^−1^ indicated the presence of the C–H species. The stretching vibration shifted to the lower wavenumbers of 3379 cm^−1^ and 3375 cm^−1^ for ZnO and Ag/ZnO, respectively, due to the H-bond formation of the O–H group in the phenolic compounds, which were present in traces in the Ag/ZnO samples [Fig. 3(c) and (d)]. In the spectra of synthesized Ag/ZnO NPs, weak bands corresponding to O–H in-plane bending and O–H phenolic group out-of-plane bending vibrations were observed at approximately 1420 cm^−1^ and 1010 cm^−1^, respectively, as depicted in Fig. 3(d).^43^ The most important FTIR data demonstrating the successful synthesis of ZnO NPs and Ag/ZnO NPs was the characteristic stretching peak of Zn–O at approximately 496 cm^−1^.^44^

### 3.3 X-ray diffraction analysis

**Fig. 4:**
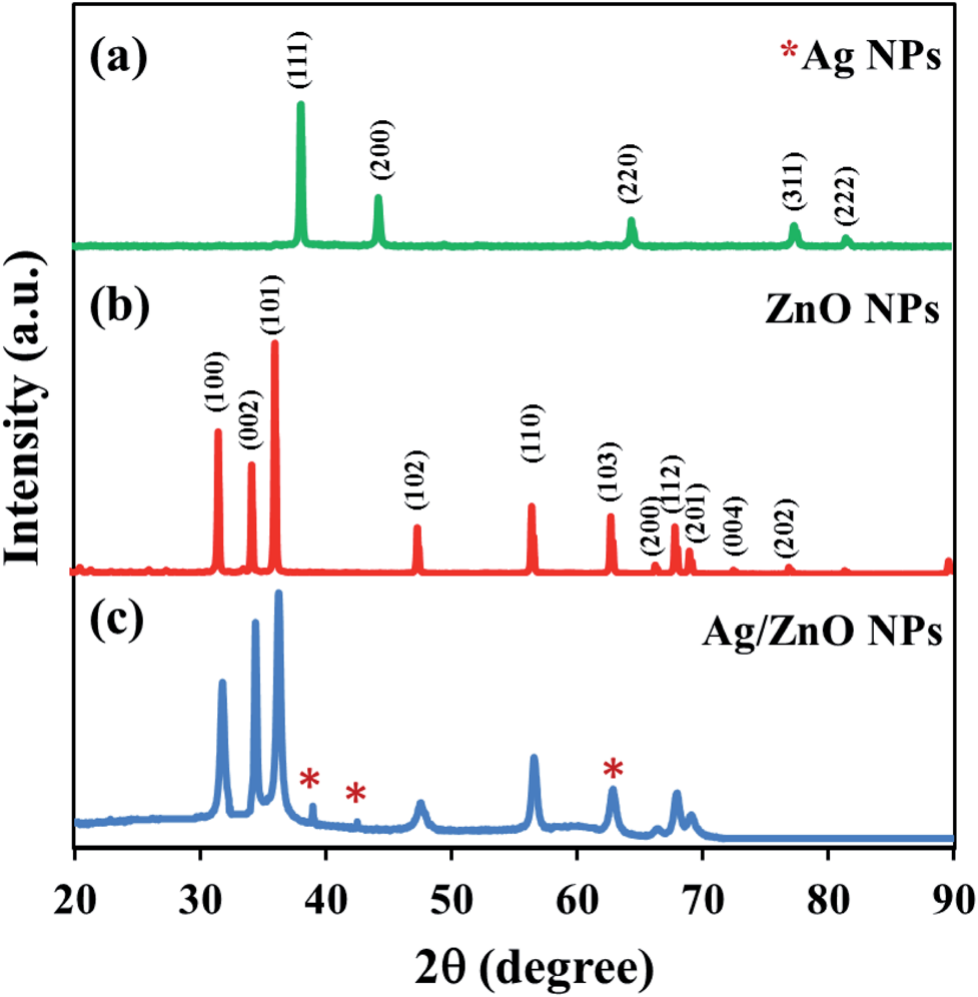
XRD patterns of (a) Ag NPs (b) ZnO NPs and (c) Ag/ZnO NPs synthesized using star fruit extract.

The crystalline structure of the synthesized nanoparticles was studied using the X-ray diffraction technique. The XRD patterns of ZnO NPs, AgNPs, and Ag/ZnO NPs were also compared. Fig. 4 depicts the crystalline structure of the nanoparticles biosynthesized under microwave irradiation at pH 10. The XRD pattern of AgNPs exhibited distinct characteristic diffraction peaks [Fig. 4(a)] at 37.34 (111), 43.54 (200), and 64.52 (220), with a face-centered cubic structure (JCPDS no. 04-0783). In the XRD pattern of undoped NPs depicted in Fig. 4(b), the appearance of intense peaks at the diffraction angles of 31.69°, 34.35°, 36.18°, 47.56°, 56.54°, 63.00°, 68.05°, and 77.04°, corresponding to the (100), (002), (101), (102), (110), (103), (200), and (202) planes, respectively, demonstrated the crystalline nature of ZnO NPs. No diffraction peak changes were detected in the prepared nanocomposites, indicating that the crystalline structure of ZnO NPs was successfully conserved as the hexagonal wurtzite phase (JCPDS card no. 36-1451).^45^Fig. 4(c) depicts the XRD pattern of Ag/ZnO NPs, in which three additional diffraction peaks were detected with the values of 38.09, 44.38, 64.51, respectively, which were indexed to (111), (200), and (220), respectively. This indicated the face-centered cubic structure of Ag and was consistent with the XRD data obtained for the synthesized Ag nanoparticles depicted in Fig. 4(a). Most of the peaks represented a lowered diffractogram of Ag/ZnO NPs, indicating that the element's structural property was reduced, which could be attributed to the generation of faults due to the doping of Ag ions.^46^ No evident peak of other impurities appeared.

### 3.4 SEM and EDX analysis

**Fig. 5:**
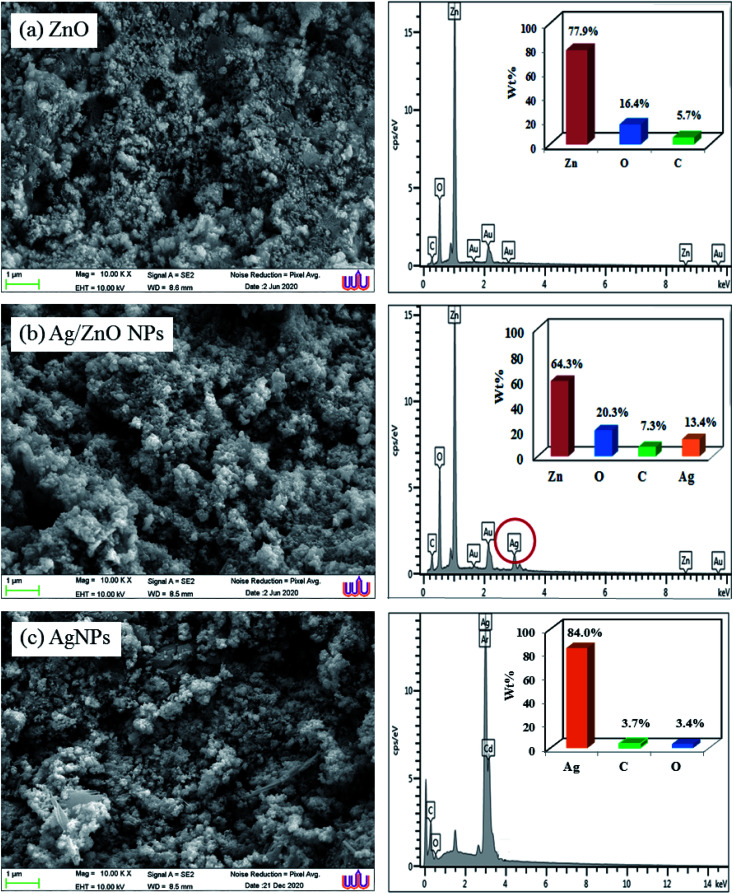
SEM images and Energy-dispersive X-ray spectroscopic spectra of the synthesized undoped ZnO NPs, Ag-doped ZnO NPs, and Ag NPs.

**Fig. 6:**
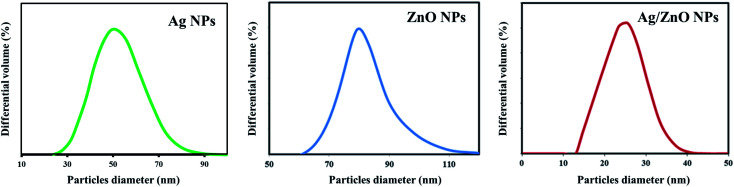
LPSA spectra of the synthesized undoped ZnO NPs and Ag-doped ZnO NPs.

Scanning electron microscopy was employed to study the surface morphology of synthesized ZnO NPs and Ag/ZnO NPs (Fig. 5). The LPSA spectrum exhibited spherical morphology with a dispersed particle size of 70–90 nm, as visible in the SEM images of synthesized ZnO NPs (Fig. 6). In the presence of Ag, the surface morphology of ZnO included smaller spherical nanoparticles compared to those in the undoped ZnO. The particle size distribution was in the range of 20–30 nm, which is also smaller compared to the synthesized AgNPs (30–70 nm). This could be due to the segregation of Ag at the nanocrystalline grain boundary. Since ZnO inhibits the nanocrystal development of ZnO, Ag-doped ZnO nanocrystals were significantly smaller than the undoped nanocrystals.^47^

The peaks of zinc, oxygen, and carbon appeared in the energy dispersive X-ray spectrum of ZnO NPs, indicating that the ZnO was devoid of contaminants. The absence of any additional peaks in the EDX spectrum indicated that pure zinc oxide nanoparticles had formed. The EDX spectra of ZnO samples exhibited strong EDX peaks at 0.3 keV, 0.5 keV, and 1.0 keV, corresponding to the emissions from the K-shell of carbon (5.70%), K-shell of oxygen (16.4%), and L-shell of zinc (77.9%), respectively. The EDX spectrum of Ag/ZnO NPs appeared similar to that of the Ag element spectrum with 13.4% weight. Moreover, the EDX spectrum exhibited a carbon peak of 7.30%, oxygen 20.3%, and L-shell of zinc 58.9%. These results indicated the successful green synthesis of Ag-doped ZnO NPs using the microwave-assisted approach.

### 3.5 TEM analysis

**Fig. 7:**
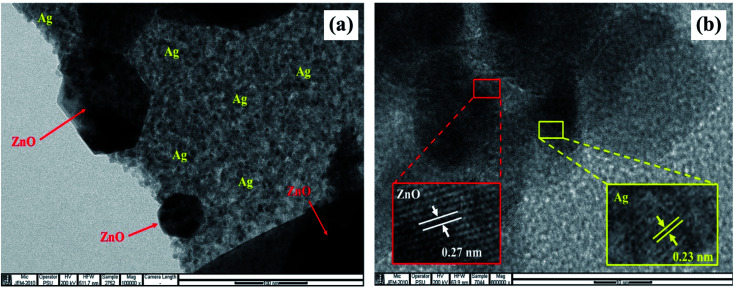
TEM images of the Ag/ZnO nanocomposite at (a) 100 nm and (b) the fringes at 10 nm scale.

TEM images of Ag/ZnO nanocomposites are depicted in Fig. 7. On ZnO surfaces, AgNPs were distributed uniformly without aggregation. A careful investigation of the images depicted in Fig. 7(a) and (b) revealed that Ag and ZnO NPs were in close contact with each other. Moreover, the TEM image depicted in Fig. 7(b) revealed that the fringes between ZnO and AgNPs had maintained their continuity. The (002) plane of ZnO NPs was represented by the 0.27 nm *d*-spacing in the lattice fringes, while the (111) plane of the face-centered cubic lattice structure of AgNPs was represented by the 0.23 nm *d*-spacing.^48^

### 3.6 Surface morphology and mapping analysis of the prepared cotton fabrics

**Fig. 8:**
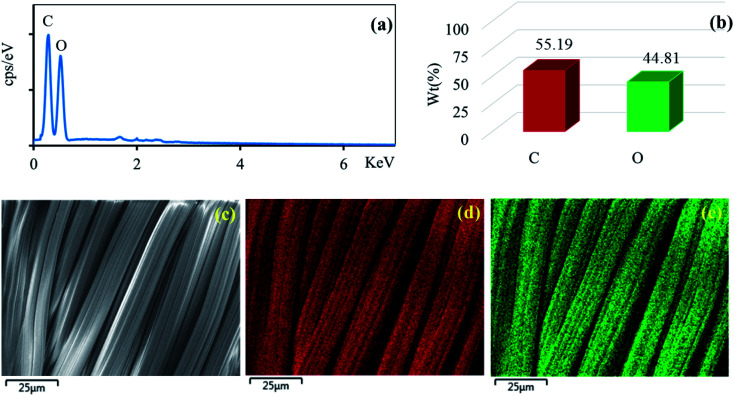
EDX spectrum (a) element component (b) of the unmodified cotton fabric and SEM image of the area (c) general elemental map and the corresponding element distribution of (d) C and (e) O.

**Fig. 9:**
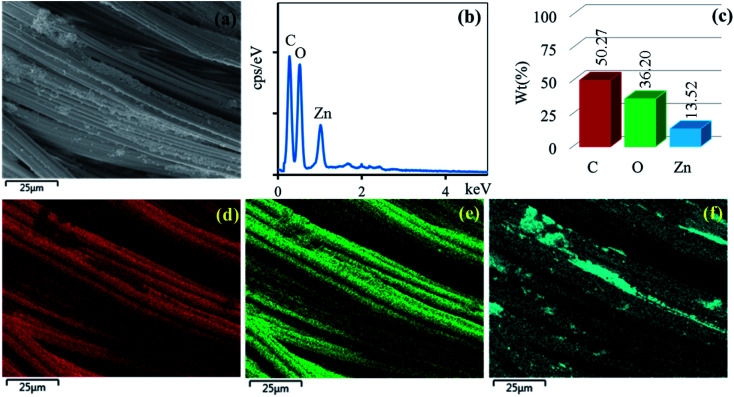
SEM image of the area (a) general elemental map and the corresponding element distribution of (d) C, (e) O, (f) Zn and EDX spectrum (b) and element component (c) of cotton fabric treated ZnO.

**Fig. 10:**
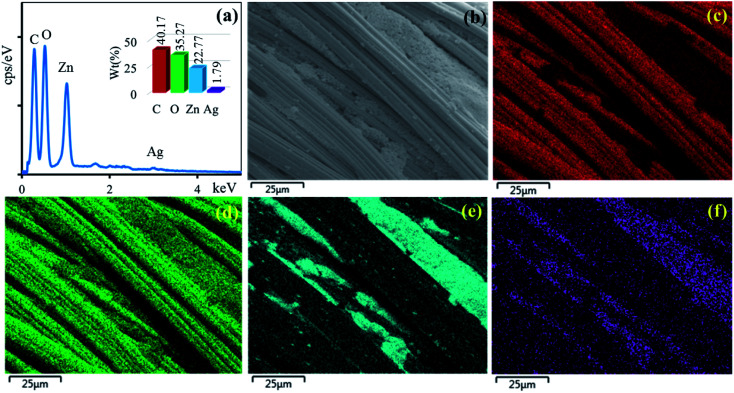
EDX spectrum (a) of cotton fabric treated Ag/ZnO and SEM image of the area (b) general elemental map and the corresponding element distribution of (c) C, (d) O, (e) Zn and (f) Ag.

The surface morphologies of the untreated cotton fabric and cured cotton fabrics treated with ZnO NPs and Ag/ZnO NPs were examined using SEM. The SEM images are depicted in Fig. 8–10. The elemental distributions of the samples were further examined through energy dispersive X-ray (EDX) analysis. The C and O elemental components of the cotton fabric are depicted in Fig. 8(a) and (b). The SEM images of the unmodified cotton fabrics depicted in Fig. 8(c) exhibits a clean and smooth surface of pristine cotton fibers with a few natural patterns. The X-ray mapping analysis images depicted in Fig. 8(d) and (e) present the C and O constituents of the cotton fibers. Fig. 9(a) revealed that the ZnO nanoparticles were coated on the cotton fibers. The Zn and O element compositions that appeared in the EDX data are depicted in Fig. 9(b) and (c). The mapping analysis images depicted in Fig. 9(d)–(f) present the C, O, and Zn distributions on the cotton fibers, respectively. The elemental compositions of Zn, O, and Ag-treated cotton were investigated through EDX, and the results are presented in Fig. 10(a). The Ag/ZnO nanocomposite was evenly dispersed throughout the cotton fibers, with a few aggregates visible in the SEM image Fig. 10(b) and X-ray mapping analysis image [Fig. 10(c)–(f)], indicating that the synthesis procedure's multilayered coating had a high degree of homogeneity. The antibacterial properties of the prepared cotton fabrics were influenced by their respective Ag/ZnO NPs coating.

### 3.7 Antibacterial activity analysis and proposed mechanism

**Fig. 11:**
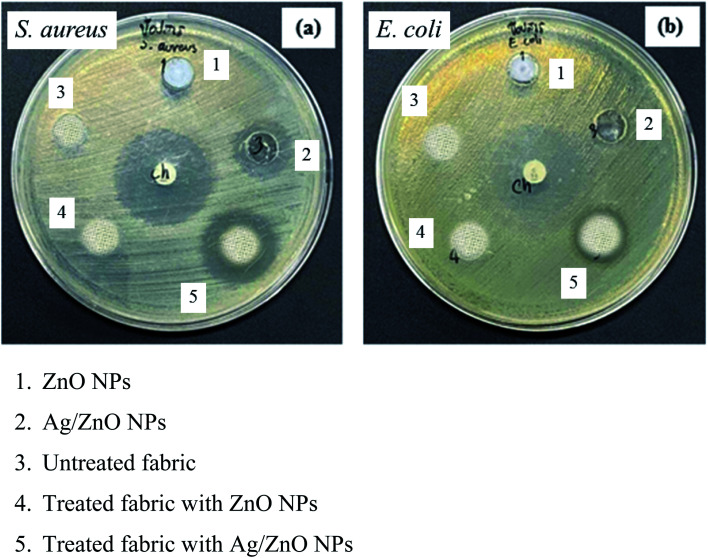
Antibacterial activity of the materials, untreated fabric, and treated fabric against (a) *S. aureus* (b) *E. coli*.

**Fig. 12:**
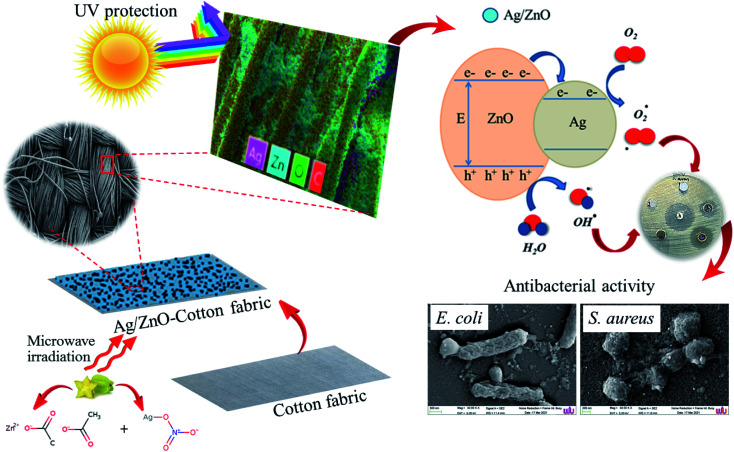
Proposed mechanism the cotton fabrics treated with Ag/ZnO nanocomposite for antibacterial activity.

The antibacterial property of the fabrics coated with ZnO and Ag/ZnO nanocomposite was determined based on the zone of inhibition. The results are depicted in Fig. 11. No evident zone of inhibition appeared for the untreated fabrics, while the fabrics treated with the synthesized Ag/ZnO NPs exhibited a zone of inhibition against both *E*. *coli* and *S. aureus*, with diameters of 36.24 ± 2.08 and 55.48 ± 2.52 nm, respectively (Table 1). The reduced crystallite size (Fig. 6) is another explanation for the antibacterial activity of the nanoparticles containing Ag. An increase in the active surface area of active nanoparticles leads to an increase in their biocidal activity.^49^ Many antibacterial mechanisms have been proposed, including nanoparticle interactions with bacteria that cause bacterial cell damage and the formation of reactive oxygen species (ROS).^50^ The proposed mechanism of ROS produced could be explained in Fig. 12. The formation of hydrogen peroxide at the surface of zinc oxide has been proposed by Sawai^51^ and Yammato^52^ as an effective technique for inhibiting bacterial growth. The number of ZnO nanoparticles per unit volume of powder is expected to increase as the particle size decreases, resulting in a greater surface area and higher hydrogen peroxide formation. Silver also has antibacterial properties.^53^ Silver nanoparticles are reported to infiltrate the bacterial cell membrane, causing significant cell damage.^54^ Besides, changes in the reactivity of the cell walls of the two bacterial species that were exposed to the Ag/ZnO nanoparticles should also be considered. The antibacterial activity was higher in the presence of *S. aureus* than in the presence of *E. coli*. The content and the structure of cell wall of Gram-positive and Gram-negative bacteria account for this disparity. Gram-negative bacteria have lipids, proteins, and lipopolysaccharides (LPS) in their cell walls, which defend them better against the biocides compared to Gram-positive bacteria.^55^

**Table 1:** UPF classifications and the levels of relative transmittance and protection

UPF range	Protection category	Effective UVB_eryt_, transmission (%)
<15	Insufficient protection	>6.7
15–24	Good protection	6.7–4.2
25–39	Very good protection	4.1–2.6
40–50, 50+	Excellent protection	≤2.5

Table 2 presents the data of the antibacterial activity of materials, untreated fabric, and treated fabric against the two bacterial species evaluated. The untreated fabrics exhibited no antibacterial activity, while the fabrics treated with Ag/ZnO nanoparticles exhibited a zone of inhibition, reflecting their antibacterial activity.

**Table 2:** Antibacterial activity of materials, untreated fabric, and treated fabric against two bacterial species^a^

Sample	Zone of Inhibition (mm) ± SD	
	*S. aureus*	*E. coli*
(1) ZnO NPs	N	N
(2) Ag/ZnO NPs	26.21 ± 1.53	13.02 ± 1.00
(3) Raw fabric	N	N
(4) Treated fabric with ZnO NPs	N	N
(5) Treated fabric with Ag/ZnO NPs	55.48 ± 2.52	36.24 ± 2.08

a N = No inhibition zone.

### 3.8 Stability of Ag/ZnO nanocomposite coated fabrics

**Fig. 13:**
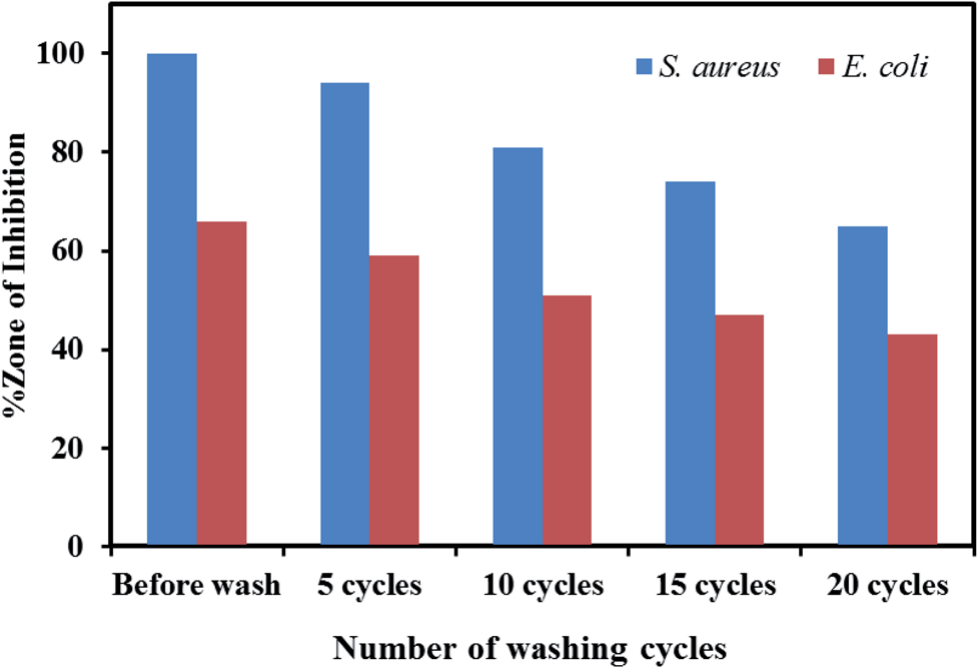
Wash durability analysis results for the cotton fabrics treated with ZnO nanoparticles and Ag/ZnO nanocomposite.

The antibacterial activity analysis indicated that only the Ag/ZnO nanocomposite-coated fabrics were efficient against Gram-positive and Gram-negative bacteria. Therefore, the fabrics coated with Ag/ZnO NPs were analyzed for their antibacterial activity after every 5 washes until 20 wash cycles. Fig. 13 presents the wash durability analysis results for the treated cotton fabrics. The fabrics coated with Ag/ZnO nanocomposites performed better against *S. aureus* than against *E. coli*. Moreover, these fabrics effectively maintained their antibacterial activity even after washing. While 5 times, 10 times, 15 times, and 20 times washing decreased the antibacterial efficiency of these treated fabrics compared to the value prior to washing by approximately 1% against *S. aureus* and 2% against *E. coli*, the antibacterial ability was retained even after 20 washes, which corresponding to research.^56^ The findings demonstrated that fabrics coated with Ag/ZnO nanoparticles were durable, as evidenced by their antibacterial activity after numerous washings, exceeding earlier studies.^57^

### 3.9 UV protection analysis

The UPF values for the control cotton fabric, ZnO-coated cotton fabric, and Ag/ZnO-coated cotton fabric are presented in Table 3. An initial analysis of the UV protection ability of the control cotton fabric revealed that it provided insufficient UV protection with a UPF of only 7.33 ± 0.58. ZnO nanoparticles and the Ag/ZnO nanocomposite greatly increased the UV-protection ability of the cotton fabric, as evidenced by the UPF values of 37.67 ± 0.58 for the ZnO-coated fabric and 69.67 ± 1.53 for the Ag/ZnO-coated fabric. These values indicate good UV protection and excellent UV protection, respectively. Silver exhibits a higher level of UV protection compared to zinc oxide. Therefore, the UPF index increases as the silver concentration increases.^58^

**Table 3:** UPF values for untreated and treated fabrics

Samples	UPF* (±SD) value	Protection value
Uncoated cotton fabric	7.33 ± 0.58	Insufficient protection
ZnO NPs coated cotton fabric	37.67 ± 0.58	Very good protection
Ag/ZnO NPs coated cotton fabric	69.67 ± 1.53	Excellent protection

## 4. Conclusions

The Ag/ZnO nanocomposite was prepared in solution for the first time using the microwave irradiation approach and *Averrhoa carambola* fruit extract, which reduced the Ag present on the surface of dispersed ZnO nanoparticles. The XRD analysis revealed that the composite structure comprised a crystalline face-centered cubic Ag phase and a hexagonal wurtzite type ZnO phase. The UV protection properties of the cotton fabrics treated with Ag/ZnO nanocomposite were excellent. The modified cotton fabrics exhibited better inhibition of Gram-positive bacteria (*S. aureus*) compared to Gram-negative bacteria (*E. coli*). The findings of this study will aid in the development of practical cotton textiles in community enterprises in Nakhon Si Thammarat Province, Thailand, allowing them to have better and more valuable properties.

## Conflicts of interest

The authors have declared no conflict of interest.

## Supplementary Material
